# Congenital Cataracts in Preterm Infants: A Review

**DOI:** 10.7759/cureus.40378

**Published:** 2023-06-13

**Authors:** AlJawhara Al-Damri, Horia M Alotaibi

**Affiliations:** 1 Ophthalmology, King Saud Medical City, Riyadh, SAU; 2 Ophthalmology, Ministry of Health, Riyadh, SAU

**Keywords:** cataract surgery, retinopathy of prematurity (rop), preterm children, preterm infant, congenital cataract (cc)

## Abstract

A congenital cataract is one of the most treatable causes of visual impairment during infancy. Preterm infants who are born alive before 37 weeks of pregnancy need special care, including proper age documentation, preoperative assessment, and monitoring postoperatively for at least 24 hours. Management of cataracts in preterm infants is critical as regards the timing of cataract surgery and the challenges associated with cataract surgery and posterior segment management for retinopathy of prematurity (ROP). This narrative review aims to provide comprehensive insight and up-to-date clinical research findings regarding the pathophysiology and management of congenital cataracts in preterm infants.

## Introduction and background

A cataract is one of the most common treatable causes of visual impairment in infancy. The prevalence of cataracts is 1.2-6.0 cases per 10,000 infants [1]. It is estimated that 15 million infants are born preterm annually [2]. Preterm infants are born alive before the 37 weeks of pregnancy are completed. The World Health Organization (WHO) developed the most widely accepted classification for preterm infants [3]. This classification further classifies preterm infants as extremely preterm (<28 weeks), very preterm (28-32 weeks), and moderate to late preterm (32-37 weeks). Preterm infants with low birth weight are at risk of developing cataracts, and an incidence of 0.97%-1.9% is estimated [4]. Also, they might develop severe visual impairment due to hypoglycemia, hypoxic-ischemic encephalopathy, and other metabolic imbalances [5]. These processes can impact the anterior visual pathway and cause cortical visual impairment, optic atrophy, optic nerve hypoplasia, or abnormal optic disc cupping. Other ocular abnormalities include strabismus, nystagmus, and ocular motor abnormalities such as tonic downward gaze and abnormal saccades and pursuits [5].

This review aims to discuss congenital cataracts in preterm infants and provide a review of the mechanism of cataracts and the relationship between cataract formation, prematurity, and previous retinopathy of prematurity (ROP). Also, this article aimed to summarize the most recent recommendations on special considerations for operating on the preterm infant in terms of anesthesia, preoperative, intraoperative, and postoperative care, along with the challenges that can be faced while managing them. A literature search was performed using the Cochrane Library, PubMed, and Google Scholar with no language or study type restrictions. The keywords used were "congenital cataract," "preterm infant," "preterm children," "retinopathy of prematurity (ROP)," and "cataract surgery." Titles and abstracts were screened for relevance.

## Review

Demographics of full-term and preterm births

Approximately 15 million infants are born preterm each year [2]. Based on results from 184 countries, preterm delivery affects 5% to 18% of all infants born. The preterm birth rate increased from 9.8% (8.3-10.9) in 2000 to 10.6% (9.0-12.0) in 2014. A total of 14.84 million preterm births occurred in 2014, the majority of which (81·1%) occurred in Asia and sub-Saharan Africa [2]. It is estimated that 70% to 80% of preterm births are spontaneous [6]. The terms gestational age, corrected age, postmenstrual age, and postconceptional age have often been misapplied in literature [7].

Gestational age and conceptional age

The inconsistent use of terminology in the literature limits the accuracy of interpretations regarding health outcomes in newborn infants, especially for those conceived using assisted reproductive technology or born preterm. Gestational age (GA), sometimes referred to as menstrual age, is defined as the time between the first day of the last normal menstrual period and the day of delivery [6,8]. The first day of the last menstrual period occurs approximately two weeks before ovulation and around three weeks before implantation of the blastocyst. Conceptional age (CA) is the true age of the fetus and represents the length of pregnancy starting from the time of conception. Gestational age is a helpful tool to monitor pregnancy, as most women know when their last period began but not when ovulation occurred. Thus, gestational age is a reliable method for estimating the expected delivery date, provided that the menstrual dates are remembered accurately [9].

Prevalence of congenital cataracts worldwide

Congenital cataract (CC) describes the lens's opacity at birth or an early childhood stage [10]. Cataracts diagnosed within the first 12 months are called infantile cataracts [11]. It is estimated that 200,000 children are blind bilaterally due to cataracts. Partial cataracts can also progress and cause visual difficulties with the child's increasing age [12-13]. Based on a meta-analysis of 27 eligible studies published from 1983 to 2014, the pooled prevalence of CC was found to be 4.24 per 100,000 cases, making CC a rare disease according to WHO standards. Results showed an increasing trend in the prevalence of CC through 2000, and Asia had the highest prevalence of CC. Furthermore, CC tended to be bilateral (54.3%), isolated, and hereditary with nuclear or total morphology. Total (31.2%), nuclear (27.2%), and posterior subcapsular (26.8%) CC were the three most prevalent types [13-15].

Prevalence of cataracts in preterm children

Several studies attempted to investigate risk factors associated with the development of CC. The national registries in Denmark were used to identify all children aged 0-17 years old born in Denmark from 1973 to 2001 to explore maternal, demographic, prenatal, and perinatal risk factors for idiopathic congenital and infantile cataracts (CIC). Results showed that preterm birth (< 37 weeks of gestation) showed a statistically significant association with the development of CIC, with infants born before 33 weeks of gestation four times more likely to develop bilateral CIC. The authors further analyzed the results and showed that such an association would most likely be explained by birth weight [16]. Haargaard and colleagues reported that the incidence rate for congenital cataracts (per 100,000 cases) in children aged 0 to two months increased from 29.9 in 1980-1984 to 55 in 1995-2000 [17].

In one study, low birth weight, a marker of prematurity in newborns, was strongly linked to the development of congenital cataracts. The authors attributed such an association to the routine screening for retinopathy of prematurity (ROP) that is usually performed among preterm infants [18]. Thus, premature infants are more likely to be diagnosed earlier in life than those that do not require routine screening for ROP, and the association of prematurity with the incidence of CC may be explained by the standard screening that is necessary for such cohorts due to their lower birth weight [19]. Several cases of congenital cataracts were also reported in preterm births [20-21]. Nonetheless, high-quality studies reporting the prevalence of CC in preterm infants are lacking.

Risk of cataracts in retinopathy of prematurity (ROP)

ROP incidence has increased in these preterm infants as a result of the high survival rate and inadequate health care regulations about screening rules and neonatal care. These infants may develop cataracts naturally or due to procedures used to treat the underlying retinal abnormality, such as laser or vitreous surgery [4]. Hong et al. conducted an epidemiological study investigating the ophthalmic complications of ROP after preterm birth using population-based databases in South Korea. They demonstrated that the total number of patients who got cataract surgery was 14 (13 cases in the first year, one case at the age of six). It indicated that 5.07% of the patients who underwent ROP intervention underwent cataract surgery throughout the 10-year follow-up. In addition, they had a higher risk of ocular complications such as amblyopia, nystagmus, glaucoma, strabismus, and retinal detachment [22]. On the other hand, Davitt et al. conducted a randomized controlled trial that revealed a few cases of early treated ROP and conventionally managed ROP that developed cataracts after diode laser, suggesting that cataract formation may proceed even without laser therapy, even if there are no observable intraoperative problems [23].

Pathophysiology of cataracts in preterm and retinopathy of prematurity (ROP)

According to Alden et al., the incidence of transient cataracts in low-birth-weight preterm infants is 2.7%. These cataracts mostly clear gradually within one month, varying between seven and 138 days, and some might progress to fully developed cataracts [24]. However, the exact cause is unknown; fluid vacuoles inside the lens point to an osmotic factor as the most likely causative agent. Prematurity alone, in the opinion of Pike et al., is an improbable cause of cataracts. According to their hypothesis, perinatal problems are frequently linked to maternal or fetal abnormalities, increasing the likelihood of cataracts developing [25].

The pathophysiology of cataracts in ROP may be related to various etiologies, as stated below:

Systemic Factors

Preterm infants are at risk for metabolic acidosis and sepsis, which can result in osmotic alterations in the lens that cause cataract formation. The typical form in these conditions is the formation of bilateral clear vacuoles along the apices of the posterior surface of the lens. After several months, these opacities gradually become less visible, transitory, and mostly clear [1, 26-28].

Complicated Cataract

A complicated cataract is present in patients with retina detachment (RD), most often in conditions of advanced-stage retinopathy due to anatomical alterations from prematurity and prior vitreoretinal surgeries [29]. It could manifest as a partially absorbed membranous or posterior subcapsular cataract with a polychromatic luster [4].

ROP Management

Numerous management modalities for ROP have been implicated, including intravitreal anti-vascular endothelial growth factor (VEGF), laser therapy, and pars plana vitrectomy (PPV). Therefore, the association of cataract formation with management is variable in the literature and needs to be investigated. The prevalence of cataracts in ROP that has undergone laser treatment varies, ranging from 0.003 to 6% [23,28]. The most secondary cataract formation occurred with the argon laser [28-29]. The hypothesized risk factors for cataract formation following laser therapy include the presence of a prominent anterior vasculosa lentis, unintentional burns to the iris or ciliary body, and confluent laser therapy.

Moreover, a thermal injury might play a role as the laser's type, power, and duration affect the total energy utilized. This is particularly evident in the case of persistent tunica vasculosa lentis (TVL), which is frequently present in premature infants. The incidence of cataracts was shown to be reduced when using diode laser energy because hemoglobin absorbs longer wavelength energy (810 nm) less [30]. On the other hand, the study by Davitt et al. revealed no relationship between laser energy and cataract [23]. Additionally, uveal effusion can lead to anterior rotation of the ciliary body and shallow anterior segment (AC), which may cause corneal-lenticular apposition and cataract formation in preterm infants [30]. In order to further reduce the likelihood of cataract formation, the df-Nd:YAG laser may be used with the proper technique, reduced cumulative energy, and avoidance of excessive indentation [4].

The lens is thicker than other ocular structures in preterm infants, and the pars plana are not formed [31-32]. Therefore, when injecting these infants with anti-vascular endothelial growth factor (VEGF), the needle tip is often pointed posteriorly toward the optic disc and 1.75 mm from the limbus. Furthermore, because the sclera in infants is more flexible than in adults, localized distortion at the injection site may result in zonular tension and harm to the posterior capsule. The SAFER-ROP protocol has updated anti-VEGF injection safety guidelines for children with ROP [33].

The risk of cataracts in infants treated for ROP

Cataract formation is seen in infants post-intervention of ROP at different periods, in laser-treated eyes ranging from 10 days to 13 months, and in post-vitrectomized eyes ranging from two months to 5.6 years [23,28,34-36]. Lambert et al. conducted a retrospective analysis of eight consecutive newborns with dense cataracts following bilateral laser photoablation for retinopathy of prematurity. They concluded that poor visual prognosis is linked to a dense cataract that forms in an infant's eye following laser photoablation. The clinical constellation seen is most aligned with anterior segment ischemia [37]. Paysse et al. suggest that the risk of acquired cataracts after transpupillary diode laser photocoagulation for threshold retinopathy of prematurity is low; it is safer than argon laser photocoagulation [38]. Khokhar et al. reported a case of bilateral cataracts in eyes with aggressive posterior ROP after administration of intravitreal bevacizumab bilaterally in an infant without evidence of touching the lens. However, the particular mechanism of cataract formation is unclear [39]. Moreover, Chandra et al. reported a case of a neonate who developed bilateral total cataract after diode laser photoablation of the avascular retina in both eyes (150 MW power, 150 ms duration, and nearly 3000 spots) for aggressive posterior ROP [40].

Timing of cataract surgery in preterm children

Early identification and surgical intervention within six to eight weeks of birth to manage CC are associated with a good prognosis [41]. After birth, eye development requires two to five months, while emmetropization of the eye is achieved by nine years of age [42]. Thus, surgical intervention is indicated for patients with visually significant greater than 3 mm central opacity or posterior pole centrally obscuring associated with strabismus or nystagmus [43]. For unilateral cataracts, cataract extraction should be performed at six weeks, while a period of eight weeks is acceptable for bilateral cataracts [44]. In circumstances where the retina is hazily visible, displaying the early stage of ROP but at risk of progression, and laser surgery cannot be done, intravitreal anti-VEGF can be injected until the cataract operation is performed. In the fourth and fifth stages of ROP, which are associated with cataracts or where the retina is close to the posterior capsule, combined lensectomy, and vitrectomy are needed to obtain optimal results [4].

General anesthesia risk in preterm children 

General anesthesia is required for the management of CC. The immaturity of preterm infants’ organs and thermoregulation places them at higher risk of general anesthesia [45]. The risk associated with anesthesia is higher in preterm infants than in full-term infants due to their immature organ systems. Respiratory function is considered a challenge because surfactant levels are inadequate, especially in infants born before 34 weeks of gestation. Premature infants also have higher oxygen consumption, tracheomalacia, and low residual pulmonary functional residual capacity [45]. Rozema et al. conducted a retrospective review to determine the incidence of apnea in preterm and term infants after deep sedation (DS) compared with general anesthesia (GA). They revealed that no apneic events in preterm or term infants were recorded after DS. In contrast, 1.7% of infants had apneic events after GA. All events occurred within two hours of monitoring in recovery [46]. Thus, postoperative observation with cardiorespiratory monitoring after the operation is required in preterm infants younger than 50 to 60 weeks of gestational age. Particularly, apneas lasting longer than 20 seconds may be associated with bradycardia and cyanosis. These infants have broncho-dysplasia, and they frequently cause problems during the first extubation after surgery [47]. Monitoring is also required for 23 hours in term infants younger than 44 weeks of postgestational age due to the increased risk for postoperative apnea [48-49].

In addition, preterm infants have immature skin, a large surface-to-body ratio, and decreased brown fat, which increases their risk for hypothermia. Thus, it is essential to maintain a warm environment during surgery [50]. Factors that increase morbidity and mortality in preterm neonates usually result from immature body systems and congenital defects. Peri/intra-ventricular hemorrhage (PVH-IVH) is also a significant cause of morbidity and mortality in premature infants [51]. The preterm neonate is at a higher risk for intracranial hemorrhage, most probably due to changes in blood flow, blood pressure, and other factors such as serum osmolality [52].

The most crucial patient characteristic that permits identifying a high-risk patient is postconceptual age (PCA), defined as gestational age plus postnatal age [53]. Numerous studies have proven that PCA is inversely related to postoperative apnea [54-59]. Optimal timing recommendations for surgery range from 44-46 postconceptual weeks [52, 60-61]. Preterm newborns less than 44 weeks postconception have been shown to have an increased risk of developing postoperative apnea. Therefore, it has been suggested that any non-essential surgery for preterm infants be postponed until they are older than 44 postconceptual weeks [60].

Recommendations for preoperative, intraoperative, and postoperative care

Preoperative Care

All preterm infants should be admitted before any procedure as a precautionary measure [59]. The following tests might be needed in exceptional cases and performed before pediatric cataract surgery under general anesthesia: glycemia, 31 transaminases, azotemia, electrolytes, blood cell count, coagulation tests, cholinesterase, urine analysis, ECG, and chest X-ray [47]. In addition, preoperative B-scan imaging is crucial to exclude the possibility of retinal detachment, especially when the posterior segment is invisible. Moreover, ultrasonography biomicroscopy (UBM) can be used to identify the site of the posterior synechiae, assess the adhesion of the posterior iris to the anterior capsule, and assess the condition of the posterior capsule for the existence of any pre-existing abnormalities and the sulcus-to-sulcus (STS) diameter [62-63].

In order to ensure successful airway management in preterm infants, it is crucial to carefully select appropriately sized equipment and medications for intubation and airway management, considering the infant's age, weight, and medical condition. Personnel responsible for airway management should receive appropriate training, and airway carts should be equipped with monitoring and backup equipment to ensure proper positioning, oxygenation, and ventilation [64-65]. When administering general anesthesia to preterm infants, it is critical to consider their weight, gestational age, and medical condition to ensure proper dosing of medications. Weight-based dosing, gestational age modification, and close monitoring are essential to minimize the risk of adverse events [66]. The pharmacological properties of medications in preterm infants can also impact dosing, requiring lower doses or longer intervals between doses for drugs with longer half-lives. These considerations are crucial to ensuring the safety and effectiveness of anesthesia for preterm infants undergoing surgery or other procedures [67].

Additionally, when performing procedures on premature infants, selecting the appropriate premedications, anesthetics, and muscle relaxants is critical. Premedications such as benzodiazepines and alpha-2 agonists are commonly used to reduce anxiety, facilitate anesthesia, and relieve pain in preterm infants before surgery [68]. Muscle relaxants such as atracurium, vecuronium, or rocuronium may also be administered to facilitate endotracheal intubation and enhance surgical conditions. However, selecting anesthetic medications for premature infant congenital cataract procedures must be carefully considered based on age, weight, and medical condition. Sevoflurane or desflurane are often used for inhalation anesthesia, while intravenous anesthetics such as propofol or ketamine may also be considered [69]. Healthcare providers can help ensure safe and effective anesthesia for premature infants undergoing surgery by choosing the most appropriate medications for each case.

In addition to anesthesia, tropicamide (0.5%-1%) and phenylephrine (2.5%) eye drops are combined to dilate premature infants. The drops should be administered twice, 10 minutes apart, one hour before surgery, as they are for ROP screening [4,70]. This is important because neovascularization of the iris is frequently seen in patients with aggressive posterior retinopathy of prematurity (APROP), which prevents the pupil from dilatation and makes it difficult to see the posterior segment and lens condition. In these conditions with significant neovascularization where the RD has been excluded, intravitreal anti-VEGF administered before cataract surgery may aid in the regression of neovascularization [4].

Intraoperative Care

Pediatric cataract surgery is challenging due to the smaller size of the eye, less scleral rigidity, more anterior capsule elasticity, and steeper anterior lens curvature. Also, the anterior segment of preterm infants differs further in that the cornea is steeper, the AC is shallower, the iris is inserted more anteriorly, and the lens is thicker and more spherical than in full-term infants [4]. As in other pediatric cases, the superior corneal incision is preferable in preterm infants. The upper eyelid shields the superior wound from trauma and exposure, thus reducing the likelihood of infection. In order to maintain the AC and have minimal fluctuations in AC depth, high-density visco-cohesive is preferred [4].

Various techniques, including mechanical stretching, sphincterotomy, and mechanical devices like iris hooks, can be tried with non-dilating pupils [4]. In addition, 0.06% trypan blue dye can be used to visualize the anterior capsule. This dye has been demonstrated to exert biochemical effects on the lens capsule, reducing its elasticity [71-72]. The ideal anterior continuous capsulorhexis size ranges from 4.5 to 5 mm [73]. It can be begun with a 26-gauge capsulotomy needle and continued with capsulorhexis forceps [74]. When the posterior capsular defect is not recognized, multiple quadrant hydrodissection can be done, making cortex removal simple and requiring less fluid. Complete lens matter removal is possible using the bimanual technique without placing unnecessary strain on the bag. Also, careful lens debris removal should be ensured to avoid postoperative inflammation [73-76].

Early intraocular lens (IOL) implantation before six months carries high-risk postoperative complications, mainly glaucoma and inflammation [77]. These preterm eyes will grow more rapidly than normal eyes, and a significant myopic shift that requires IOL exchange has been reported [78]. Also, studies found no association between axial length and refractive error in small preterm infants compared to full-term children [35,79]. Patients treated with the laser have greater axial length growth than those treated with anti-VEGF or who spontaneously regressed. These elements must be considered because these situations might require more undercorrection than normal eyes [80-81]. Primary IOL implantation is not performed in many infants; contact lens or spectacle correction is used for visual rehabilitation. Implanting secondary IOL in children at one year old is acceptable and safe, but there is considerable controversy in children younger than the age of one, and further study is required [82]. IOL insertion is possible if the corneal diameter is greater than 10 mm, the STS diameter is greater than 9 mm, the axial length is greater than 17 mm, and no other ocular characteristics prevent IOL implantation [63]. Over time, different IOL calculation formulae have been provided; however, children under two years show the most variability [76,83-85].

Postoperative Care

All postoperative preterm infants should be monitored closely for 12 to 24 hours to prevent apnea and bradycardia [59]. Post-congenital cataract surgery in preterm infants is associated with a higher risk of immediate complications due to organ immaturity and surgical techniques [86]. Given their vulnerability, preterm infants may experience respiratory depression and hypotension following anesthesia and surgery [87]. This highlights the importance of vigilant monitoring, prompt intervention, appropriate medications, and non-pharmacologic interventions, such as skin-to-skin contact, to manage postoperative pain and promote wellness [88]. In addition, preterm infants have undeveloped immune systems and prolonged hospital stays, which increase their risk of infection, making timely diagnosis and antibiotic treatment crucial for management [86]. Furthermore, the risk of postoperative hemorrhage is higher in preterm infants due to immature coagulation systems, making monitoring transfusions, hematocrit, and hemoglobin levels essential [86-88].

Moreover, opioids are not advised for postoperative analgesia in preterm and ex-preterm infants due to their severe effects on the respiratory system and propensity to result in postoperative apnea. Acetaminophen (paracetamol) is generally preferred. Most NSAIDS should not be administered to infants younger than six months [89-90]. Topical cycloplegic and steroid therapy are crucial for postoperative care [91-92]. Patients with iris manipulation and those with posterior synechiae are more likely to experience postoperative inflammation. Therefore, topical steroids are usually needed in these patients and are gradually tapered. This avoids postoperative posterior iris synechia, which could cause glaucoma and make it challenging to examine the retina thoroughly [4].

Ocular complications in preterm infants post cataract surgery

The most common complication after CC surgery is glaucoma. Although advances have been made in CC management, secondary glaucoma (SG) is a major sight-threatening complication, with open-angle glaucoma being the predominant type [51]. The benefit of early surgical interventions, which can result in better visual outcomes in pediatric cataract patients, must be weighed against the increased risk of postoperative glaucoma incidence [51-52]. However, the incidence of glaucoma after pediatric cataract surgery is very low in patients for whom an intra-ocular lens (IOL) was implanted. Patients with aphakic eyes after pediatric cataract surgery would be at higher risk of developing glaucoma, mainly if the surgery were performed before four months of age [93]. Posterior capsule opacification, pupillary membrane, inflammatory response, lens proliferation, and amblyopia are common complications of post-congenital cataract surgery. Less common adverse events include significant bleeding, infections, and retinal detachment [94]. Moreover, cataract surgery is associated with postoperative refractive errors. These include loss of accommodation, planned refractive errors based on age or eye status, unexpected refractive errors due to biometry technique accuracy, and late refractive changes due to ocular growth [95].

The outcome of cataract surgery in preterm children

A retrospective study was conducted by Yu et al., evaluating 26 eyes of 14 premature infants with or without ROP who underwent cataract surgery. Patients were followed up after the last surgery for 0.5 to 3.1 years. They revealed that the best corrected visual acuity at the most recent exam was good fixation or better than 20/80, except for two eyes: one with esotropia and the other with a dense pupillary membrane [96]. Ezisi et al. assessed the outcomes of cataract surgery in 28 eyes of 22 children with ROP [34]. The results showed that two eyes experienced intraoperative posterior capsular rupture. Postoperative complications included visual axis opacification, secondary glaucoma, IOL capture, and refractive changes similar to those in eyes without ROP. In the 23 eyes where postoperative visual acuity testing was possible, 11 had vision better than 20/200. The mean myopic shift in the 11 patients with at least a two-year follow-up was -3.07 D in pseudophakic and -8.75 D in aphakic [34].

Whether to operate on each eye individually, spacing out the surgery by some time, or concurrently under one general anesthetic is another major point of debate when dealing with bilateral cataracts in severely preterm infants. The benefits of simultaneous bilateral surgery for bilateral pediatric cataracts were reviewed by Guo et al. [97] with this technique; they observed no significant complications in 16 children representing 32 eyes. With nine children (18 eyes), Zwann et al. [98] had the same success as Totan et al. [99] with 12 children (24 eyes). The issue of bilateral simultaneous cataract surgery raising the risk of sight-threatening complications such as endophthalmitis and expulsive choroidal hemorrhages is discussed in all three of these studies. Moreover, William et al. reported a case series of four preterm infants (eight eyes) [77]. The youngest was 25 weeks, and the eldest, 32 weeks, underwent simultaneous bilateral cataract surgery. Three infants underwent primary posterior chamber IOL implantation; one had a primary lensectomy with contact lens correction. All eyes had significant myopia changes of up to -15.00 d. Also, secondary membranectomies were necessary for all six eyes with IOLs. They concluded that early IOL implantation is very effective in infants under one year with a lower risk of glaucoma. However, the significant posterior capsule opacification (PCO) risk requires a further surgical membranectomy [77].

## Conclusions

Preterm infants are a particular group of patients who need special care. Proper age documentation, mainly postconceptual age, defined as gestational age plus postnatal age, is the most accurate infant age documentation; this is a crucial factor related directly to postoperative apnea. All preterm infants should be admitted, and preoperative assessment, including basic laboratory tests, should be performed. Also, they should be monitored postoperatively for at least 24 hours, specifically for cardiorespiratory function, to avoid postoperative apnea and deterioration.

Regarding timing for cataract surgery in preterm infants, 44-46 postconceptual weeks is recommended. Intraocular lens (IOL) implantation before six months of age was associated with a risk of inflammation, posterior capsular opacification, and significant myopic shift, necessitating IOL exchange in the future. Moreover, IOL calculation for preterm infants treated with the laser must be a considerable element because of the significant myopic shift in laser-treated eyes.
